# Behaviors and Strategies of Bacterial Navigation in Chemical and Nonchemical Gradients

**DOI:** 10.1371/journal.pcbi.1003672

**Published:** 2014-06-19

**Authors:** Bo Hu, Yuhai Tu

**Affiliations:** IBM T. J. Watson Research Center, Yorktown Heights, New York, United States of America; Princeton University, United States of America

## Abstract

Navigation of cells to the optimal environmental condition is critical for their survival and growth. *Escherichia coli* cells, for example, can detect various chemicals and move up or down those chemical gradients (i.e., chemotaxis). Using the same signaling machinery, they can also sense other external factors such as pH and temperature and navigate from both sides toward some intermediate levels of those stimuli. This mode of precision sensing is more sophisticated than the (unidirectional) chemotaxis strategy and requires distinctive molecular mechanisms to encode and track the preferred external conditions. To systematically study these different bacterial taxis behaviors, we develop a continuum model that incorporates microscopic signaling events in single cells into macroscopic population dynamics. A simple theoretical result is obtained for the steady state cell distribution in general. In particular, we find the cell distribution is controlled by the intracellular sensory dynamics as well as the dependence of the cells' speed on external factors. The model is verified by available experimental data in various taxis behaviors (including bacterial chemotaxis, pH taxis, and thermotaxis), and it also leads to predictions that can be tested by future experiments. Our analysis help reveal the key conditions/mechanisms for bacterial precision-sensing behaviors and directly connects the cellular taxis performances with the underlying molecular parameters. It provides a unified framework to study bacterial navigation in complex environments with chemical and non-chemical stimuli.

## Author Summary

Bacteria, such as *E. coli*, live in a complex environment with varying chemical and/or non-chemical stimuli. They constantly seek for and migrate to optimal environmental conditions. A well-known example is *E. coli* chemotaxis which direct cell movements up or down chemical gradients. Using the same machinery, *E. coli* can also respond to non-chemical factors (e.g., pH and temperature) and navigate toward certain intermediate, optimal levels of those stimuli. Such taxis behaviors are more sophisticated and require distinctive sensing mechanisms. In this paper, we develop a unified model for different bacterial taxis strategies. This multiscale model incorporates intracellular signaling pathways into population dynamics and leads to a simple theoretical result regarding the steady-state population distribution. Our model can be applied to reveal the key mechanisms for different taxis behaviors and quantitatively account for various experimental data. New predictions can be made within this new model framework to direct future experiments.

## Introduction

Living systems detect changes in the environment and try to find optimal conditions for their survival and growth. As one of the best-studied systems in biology, bacterial chemotaxis allows bacteria (such as *Escherichia coli*) to sense chemical gradients and navigate toward attractant or away from repellent [1]–[4]. This *gradient sensing* strategy makes cells move unidirectionally toward the extreme levels of stimuli. However, for other natural factors (such as pH and temperature), the physiological optimum does not locate at the extreme but at some intermediate level in the respective gradient. To find such intermediate point, it requires a more sophisticated strategy, namely, *precision sensing*. Both pH taxis [5]–[9] and thermotaxis of *E. coli* [10]–[16] provide us inspiring examples of precision sensing.

Amazingly, *E. coli* cells use the same signaling system to achieve these different navigation tasks. Different external signals are sensed by several types of transmembrane chemoreceptors, among which the Tar and Tsr receptors are the most abundant [17]. For chemotaxis, binding of attractant (or repellent) molecules to chemoreceptors triggers their conformational changes and affects the autophosphorylation of the histidine kinase CheA [3], [4]. Analogous to ligand binding in chemotaxis, both temperature and pH affect the conformational state of chemoreceptors and hence the CheA activity. Regardless of the way of being activated, phosphorylated CheA transfers its phosphate group to the response regulator CheY in the cytoplasm. The phosphorylated CheY molecules (denoted as CheY-P) then bind to the flagellar motors, increase their probability of clockwise rotations, and cause *E. coli* to tumble. The resulted alternating run and tumble pattern can steer cells to advantageous locations. To make temporal comparisons of stimuli, a short-term memory (or adaptation mechanism) is required [4], [18], [19]. This is achieved by the slow methylation-demethylation kinetics, as catalyzed by two enzymes (CheR and CheB) that add and remove methyl group at specific sites of receptors, respectively.

How does a bacterium navigate through its environment with different chemical and nonchemical cues by using the same signaling and motility machinery? How do bacterial cells make decisions under competing chemical and/or nonchemical signals? How accurately and reliably can bacteria find their favored conditions (such as the preferred temperature or pH)? and how do they tune their preference for precision sensing? We aim to address these questions under a unified theoretical framework, given that different taxis behaviors are based on the same sensory/motion machinery.

To this end, we develop a multi-scale model which incorporates intracellular signaling pathways into bacterial population dynamics. The continuum population model reveals a simple theoretical result for the steady state cell distribution, which is found to be determined by the direction-dependent tumbling rates (transmitted through intracellular signaling pathways) as well as the dependence of the swimming speed on external factors (such as temperature). This new finding enables us to systematically analyze bacterial navigation in chemical, pH, and temperature gradients. From each application, we have made quantitative comparison with the available experimental data and have gained new insights about the mechanisms of bacterial taxis. Our general model can be extended to study bacterial migration in complex environments (e.g., with a mixture of chemical and nonchemical stimuli) and provide quantitative predictions to be tested by future population level experiments.

## Results

### The Unified Model for Bacterial Taxis: A Simple Result for Steady State Cell Distribution

Our unified model for bacterial taxis is developed on the basis of a number of previous models at different scales [20]–[29]. We incorporate microscopic pathway dynamics into the macroscopic transport equations [22], [28] and derive a closed-form solution for the steady state cell distribution in chemical and/or nonchemical gradients. In the following, we outline the main steps in obtaining this key result (Eq. 5), with more details about our model given in Text S1.

**Figure 1:**
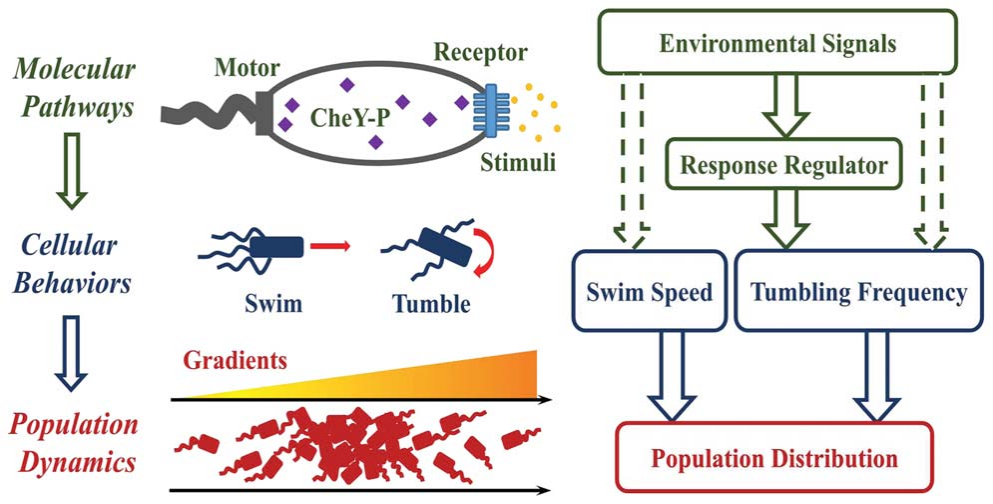
Illustration of the multiscale architecture of our unified model. Environmental signals are sensed by the transmembrane receptor-kinase complexes which controls the level of the intracellular response regulator (CheY-P). The response regulator controls the rotational direction of flagellar motors and the bacterial tumble frequency. Some environmental factors (such as temperature) can also affect bacterial swimming speed and motor switching dynamics. The population distribution of cells is finally shaped by the alternating tumble and swim behaviors.

The architecture of our model is illustrated in Fig. 1. The environmental signals (such as chemoattractants, pH, and temperature), denoted by 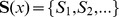, can be sensed by different types of transmembrane chemoreceptors and converted into the total receptor-kinase activity, denoted by 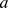. This activity represents the internal state of the cell and is described by the Monod-Wyman-Changeux (MWC) two-state model [23]–[26], [29]: 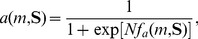
(1)where 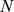 measures the degree of receptor cooperativity and 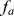 represents the free energy difference between the active and inactive receptor conformations. The total activity, 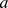, depends on the average methylation level of receptors, 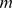, which restores 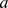 to the adapted level, 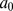, over a time scale 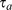. For simplicity, the methylation rate is taken to be linear in 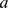 and hence the methylation dynamics can be described by: 
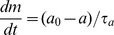
. Here, we do not distinguish the methylation dynamics for different types of receptors (which appears to be regulated by the receptor-specific activity [30]) and consider 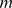 as the average methylation level of the whole receptor cluster. This treatment does not affect our main results since we are only interested in the total receptor-kinase activity, 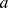, of the entire receptor cluster.

A swimming bacterial cell may change its direction due to two mechanisms: the active transition to the tumbling state and the passive rotational diffusion (characterized by the rotational diffusion rate, 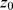). According to the measured flagellar CW bias [31], the (instantaneous) rate of going into the tumbling state can be described as: 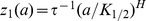, where 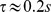 is the duration time of the tumbling state, 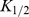 represents the activity level at which the CW bias is 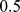, and 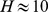 denotes the ultrasensitivity of the motor response to CheY-P. Combining these two effects, the effective tumbling rate 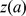 is given as: 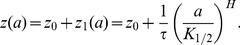
(2)


In response to environmental signals, a population of bacteria will move in the physical space. Different from purely passive Brownian particles, cells also “distribute” in the internal state space, as each cell carries its own internal activity 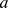 when moving around. In the one-dimensional setup, let 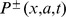 denote the probability to find a cell being in the internal state 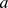 and moving in the “
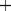
” or “
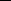
” direction at 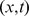. One can write down the master equation that governs the evolution of these probabilities (Text S1). As in many experiments, here we study the distribution of cells constrained in a finite chamber with a chemical or non-chemical gradient. The cell population distribution will equilibrate given enough time as the diffusion of cells balance the taxis drift effect. Using the zero-flux condition in the master equation leads to an exact expression for the steady state cell distribution 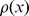 (Text S1): 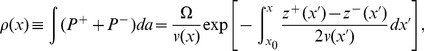
(3)where 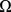 is the normalization constant and 
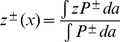
 represents the average tumbling rate for the right or left moving cells at the same position 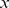.

It is clear from Eq. (3) that the cell distribution is determined by the two motility characteristics, tumbling rate (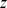) and swimming speed (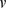). On one hand, the local cell density is inversely proportional to the local swimming speed which may depend on the external condition 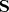. Intuitively, it is easier for cells to leave a region if cells move faster there and thus cells spend more time in regions of low swimming speed. On the other hand, the cell density also depends on the *accumulative* (integrated) effect of the tumbling rate difference between the left and the right moving cells. For example, if 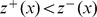, cells tend to move in the right (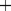) direction on average because it is more difficult for cells to enter a region where they tumble more frequently.

What is the origin of different tumbling rates (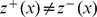) for cells moving in different directions? The tumbling frequency is controlled by the CheY-P level which is proportional to the total activity, 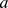. At any location 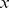, the internal activity 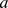 is not fixed but distributed around its average, 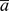. In fact, the average activity of the left moving cells (
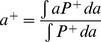
) is different from that of the right moving cells (
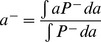
) as these two populations carry different average receptor methylation levels (memories). This activity difference can be evaluated (see Text S1 and Figure S1 for more details): 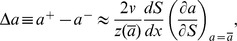 which is proportional to the (average) run length 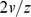 and is valid as long as the adaptation time 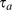 is much longer than the average run time 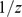. The activity difference 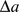 can be used to evaluate the tumbling rate difference (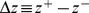): 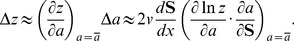
(4)


By using the above expression for 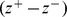 in Eq. (3), we finally obtain a simple expression for the steady state cell distribution 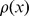: 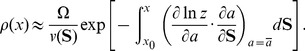
(5)


The equation for 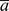 is given in Text S1.

This general expression (Eq. (5)) for the cell distribution is the main theoretical result of our paper. It shows that the steady state cell distribution is determined by two separable effects, the local effect of swimming speed 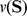 and the accumulative effect of the gradient-dependent tumbling rates governed by internal signaling dynamics. In a previous work [20], [21], a simple relation, 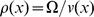, was derived by assuming that the tumbling rate directly depends on the local environment factor. This treatment, however, did not take into account the cell's internal state or memory. Therefore, although the 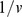 dependence in Ref. [21] agrees with our Eq. (5), the integrated effect of the intracellular signaling dynamics was not identified or captured before.

The intracellular signaling response to specific stimuli and the motor response to the response regulator are characterized by the free energy function 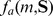, and tumbling rate 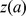, respectively. These functions can be determined by molecular and cellular level experiments, such as Ref. [26] for 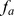 and Ref. [31] for 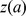. Here, our model shows how population level (macroscopic) behaviors of cells can be predicted quantitatively based on these molecular level (microscopic) signaling and response characteristics. The general expression for steady state cell distribution, Eq. (5), provides a unified framework to systematically study diverse bacterial navigation behaviors in response to different chemical and non-chemical gradients, as will be shown in the following. We will also compare our theoretical results with the available experiments and make quantitative predictions on population-level behaviors of bacteria in more complex environments.

### Population-Level Sensitivity of Bacterial Chemotaxis

We first apply our unified model to the case of bacterial chemotaxis. For simplicity, the chemical gradient (e.g. aspartate), denoted by 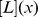, is specifically sensed by one type of receptors (e.g. Tar) whose average activity can be described by the two-state model, Eq. (1). The free-energy difference between the active and inactive states is given by [25], [29]: 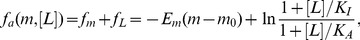
(6)where 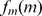 and 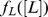 denote the methylation- and ligand-dependent contributions, respectively. The prefactor 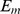 is the free-energy change per added methyl group, 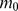 is a reference methylation level, and 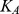 and 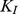 represent the dissociation constants for the active and inactive conformations, respectively.

*E. coli* swimming speed (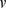) does not vary with the aspartate concentrations and is treated as constant here. By Eq. (5), it is easy to derive the steady-state cell distribution: 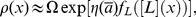
(7)with a dimensionless factor 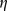 that represents the effective sensitivity of the bacterial population to the environment: 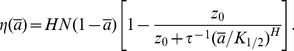
(8)


The effective sensitivity 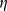 is proportional to the signal amplification factors at both the receptor and the motor levels (i.e., 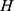 and 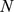). It is dampened by the rotational diffusion (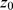), because random collision of cells with the medium reduces directed chemotaxic motion. The dependence of 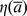 on the average activity 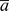 is nontrivial: on one hand, an increase of 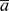 could significantly boost the intrinsic tumbling (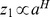) and thus suppress the negative role of rotational diffusion; on the other hand, chemoreceptors become less responsive at a higher activity level 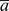. Consequently, 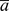 has to be in a narrow optimal range in order to achieve high sensitivity.

**Figure 2:**
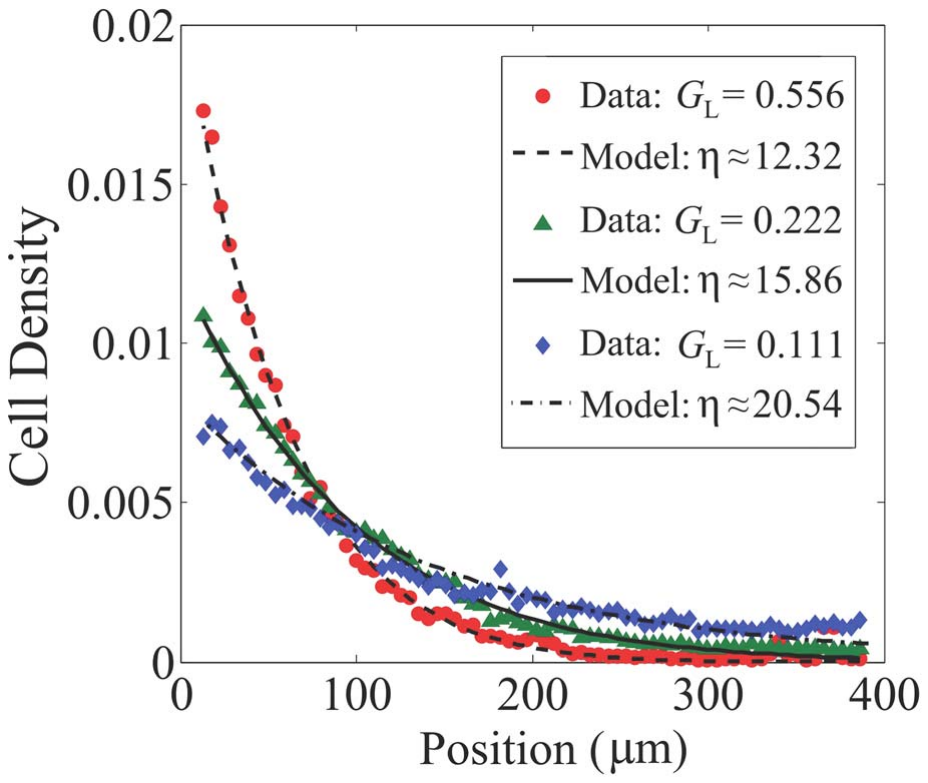
The cell density distributions for bacterial chemotaxis. Different density profiles correspond to different gradients of varying steepness 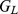. Symbols represent the experimental data from Ref. [32], whereas lines denote the fitting of our Eq. (7) to the data. We used 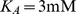, 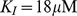 for MeAsp here.

Our model allows for quantitative comparison with experiments. As shown in Fig. 2, the same functional form given in Eq. (7) can be used to fit the cell distribution data [32] in different attractant gradients. The coefficient 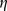 inferred from experiments (Fig. 2) appears to decrease with the gradient steepness 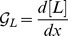, indicating a higher population-level sensitivity in shallower chemical gradients. This trend agrees with the observation in our model that the average receptor activity 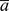 tends to increase with the gradient steepness in closed geometry. This increase in 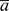 is caused by the back flow of cells due to diffusion as 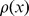 is peaked at the boundary with the higher attractant concentration (see Fig. 2). Note that this is different from the open geometry case where the cell density is constant and there is no net diffusive back flow of cells.

### Tunability vs. Accuracy of *E. coli* pH Taxis

*E. coli* can sense pH changes in the environment. According to recent experiments [8], Tar receptors exhibit an attractant response to a decrease of pH while an opposite response was observed for Tsr. The balance between the two opposing receptors leads to a preferred pH level for the wild-type *E. coli*, i.e., precision sensing, as suggested by our recent model study [9] of intracellular pH responses. Here, we examine how accurately and how robustly a population of bacteria could find their preferred pH level.

The extracellular pH modulates the receptor-kinase activity primarily by affecting the periplasmic domain of the Tar and Tsr receptors. The total receptor-kinase activity can be described by the generalized MWC model for heterogeneous types of receptors. The total free energy between the active and inactive states is given by 
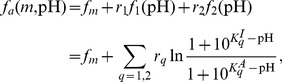
(9)where 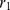 and 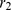 denote the fractions of Tar (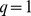) and Tsr (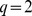) in the receptor cluster, respectively. The dissociation constants 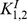 and 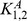 for the inactive and active receptors are expressed in the pH scale. The observed opposite responses to pH changes indicate that 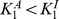 for Tar and 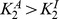 for Tsr. Without loss of generality, we set 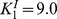 and 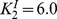 for numerical examples.

As the *E. coli* motor speed does not vary significantly with the external pH [33], we can take the swimming speed as constant here. Using Eq. (5), one can easily obtain the cell distribution in a pH gradient: 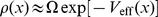, with the effective potential 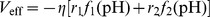. The competition between the opposing pH dependence of 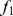 (from Tar) and 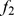 (from Tsr) leads to the accumulation of cells at an intermediate (preferred) pH level, which can be analytically determined by the condition 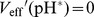; see Text S1 for more details.

**Figure 3:**
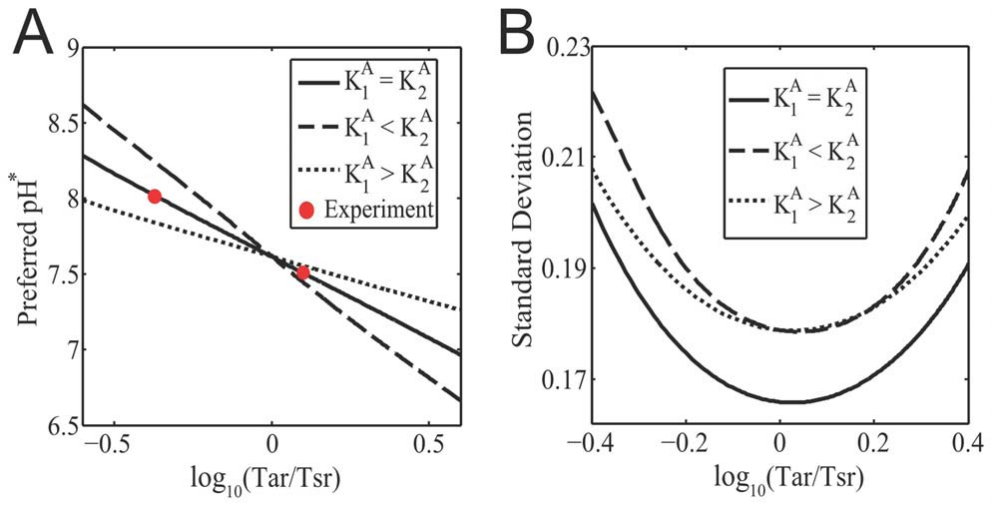
Tunability and accuracy of bacterial pH taxis. (A) The preferred pH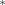 versus the logarithm of the Tar/Tsr ratio to base 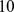 for three representative parameter regimes: 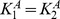, 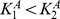, and 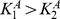. The red symbols represent the experimental data from Ref. [8] and seem to coincide with the model curve for 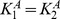. (B) The standard deviations of the cell distributions as a function of 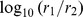 for the three representative parameter regimes: 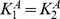, 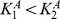, and 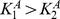. In the above numerical examples, we have fixed 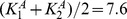, 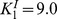 and 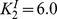.

The preferred pH is mostly sensitive to the relative abundances of receptors (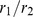) and the values of 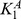 and 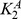. Based on our analysis and simulations, we find an empirical equation (Text S1), 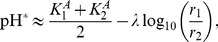
(10)which shows the logarithmic dependence of the preferred pH on the relative abundance of Tar and Tsr (Fig. 3A). The coefficient 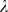 in Eq. (10) varies with the dissociation constants and can be interpreted as a measure of tunability of the preferred pH upon changing the Tar/Tsr ratio. Theoretically, this coefficient is close to one (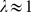) when 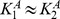 (Text S1). Numerically, we also found that higher tunability (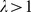) can be achieved if 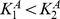 and the opposite holds for 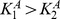 (Fig. 3A). Our results can be compared with the recent experiment [8], where the pH preference point (pH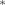) was observed to shift from 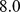 to 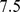 when the Tar/Tsr ratio (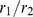) changed from 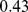 to 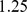 (symbols in Fig. 3A). Using these data with the empirical Eq. (10) yields 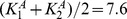 and 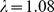, which together indicate that 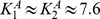.

In addition to the preferred pH, our population model also tell us the dispersion (or accuracy) of bacteria seeking and aggregating around their favored pH, which can be quantified by the standard deviation of the cell distribution in the pH scale. As shown in Fig. 3B, the dispersion measure turns out to be minimal for the scheme 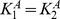, compared to the dispersion for either 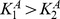 or 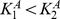. Therefore, one possible advantage for having 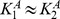 (Fig. 3A) in *E. coli* is the optimal accuracy of pH sensing at population level, though with a tradeoff of a modest tunability (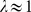) for 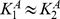.

### The Inversion of Thermal Response at the Critical Temperature

Bacteria are able to sense thermal variations and migrate toward their favored temperature [10]–[15], another example of precision sensing. However, unlike in pH sensing where two types of receptors, Tar and Tsr, respond in opposite ways to a pH change, temperature sensing can be achieved by a given type of receptor (Tar) which changes the sensing mode (from being a warm sensor to a cold sensor) as its methylation level 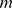 increases across a critical level 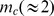 [12], [13]. Added to the complexity is the fact that temperature affects many other aspects of motility, such as the swimming speed and the motor switch sensitivity. Here, we first demonstrate how a chemoreceptor acts as a thermal sensor that inverts response at some critical temperature. In the next section, we will study how all those temperature-sensitive factors affect thermotaxis at the population level.

**Figure 4:**
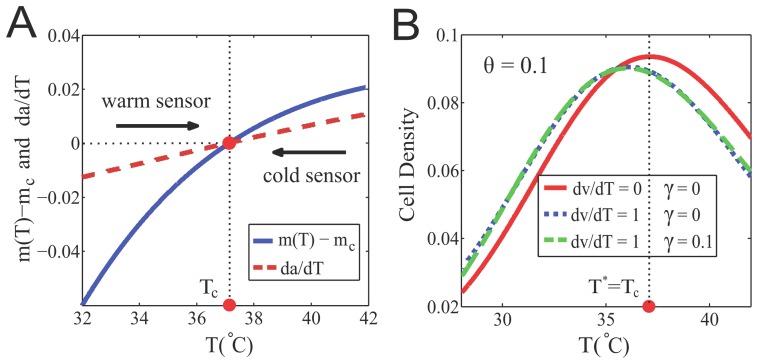
Inverted response to temperature changes and bacterial thermotaxis. (A) The steady-state methylation level subtract the critical methylation level, 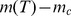, and the receptor response to temperature changes, 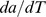, as a function of temperature. The critical temperature 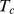 is determined by the crossing point where 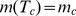 (or equivalently 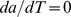). Tar acts as a warm sensor for 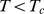 and a cold sensor for 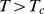, which drives the cells towards 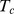 from both sides. (B) The steady-state cell distribution, 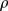, as a function of temperature. For illustrative purposes, we assume that the swimming speed, 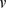, increases linearly with temperature and that the motor dissociation constant is 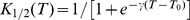, with a constant parameter 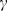. Three cases are considered. The red solid line corresponds the case where both 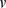 and 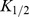 are constant; the blue dot-dashed lines is for the case of constant 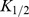 (i.e. 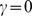) and 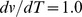; the green dashed line is generated by using 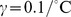 and 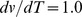. Evidently, the steady-state cell distribution can be changed by the temperature dependence of the speed 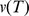, but it is insensitive to the temperature dependence of motor sensitivity 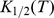. Here, we fix 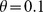 in all numerical examples. Other parameters used include: 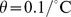, 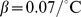, 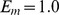, 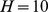, 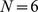, 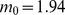, 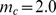, 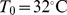, 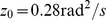, and 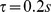.

For simplicity, we consider *E. coli* cells that only express Tar receptors and migrate in a linear temperature gradient. In general, the total free energy for the Tar activity can be described as 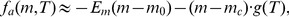
(11)where 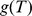 describes how temperature affects the total free energy and where 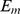 refers to the free energy change per added methyl group (in units of 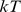) at a given reference temperature 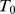 (i.e., 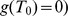. Note that a linear function 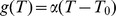 with 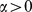 was used in a previous model of thermotaxis [16]. However, it is easy to verify that as long as 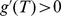, the Tar receptor switch from being a warm sensor (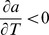) when 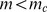 to a cold sensor (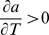) when 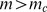; see Fig. 4A.

It is observed experimentally that the adapted activity also changes with temperature [26], [34]. This can be modeled by 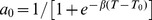. For *E. coli*, it is reported that 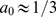 at room temperature 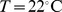 and 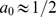 at 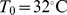, leading to an estimate of 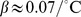. The critical temperature 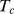 at which Tar inverts its response is defined by 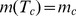. Using the condition 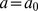, we can obtain the steady state methylation level 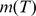 as well as the critical temperature: 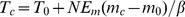, which is determined by the (upstream) receptor kinetics. This also leads to a simple relationship: 
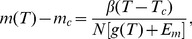
(12)showing that the average methylation level relative to 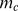 changes sign at the critical temperature 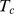 (Fig. 4A). According to Eq. (12), when 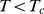, the Tar methylation level is less than 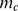 (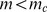), so the Tar receptor is a warm sensor driving the cell towards 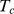 from lower 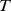; when 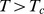, the Tar methylation level is greater than 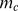 (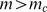), now the Tar is a cold sensor driving the cell towards 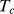 from higher 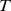. This is the basic mechanism for cell accumulation around 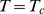.

### Two Channels Drive Bacterial Thermotaxis: Speed and Sensing

Besides the receptor-kinase activities, temperature also affects other aspects of the system. For example, it is observed for *E. coli* that both the motor dissociation constant, 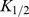, and the swimming speed, 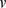, change with temperature [34], [35]. It remains unclear whether and how different temperature-sensitive factors affect the performance of bacterial thermotaxis.

Using Eq. (5), one can derive the steady-state cell distribution over the temperature range 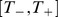: 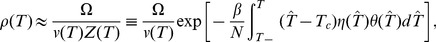
(13)where the function 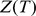 represents the effect of the direction-dependent tumbling rates governed by the thermosensory system. In Eq. (13), 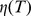 is the effective sensitivity defined in Eq. (8) and has weak dependence on temperature through both 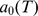 and 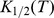. The function 
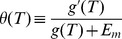
 is introduced for convenience and represents the effect of the sensory system.

Eq. (13) shows that there are two independent channels affecting bacterial thermotaxis: one is the swimming speed 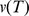, and the other is the sensory system that controls the rotational direction of flagellar motors. In contrast to the local speed effect 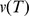 which is direct and memoryless, the sensing effect 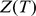 is indirect (channeled through signaling networks and motor control) and relies on the slow adaptation dynamics which encodes memory for the system to sense the environment [19], [29].

Near the critical temperature 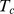, we can compute 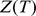 by keeping only the leading order term in 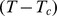, that is, 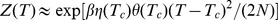. The expression for the cell density follows: 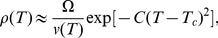
(14)where 
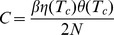
 is a positive constant when 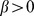 and 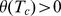. It is clear from the above equation that for a constant 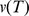 cells will accumulate around the temperature 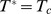 (Fig. 4B). The accumulation temperature 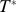 can be shifted from 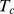 by the dependence of the swimming speed on temperature, e.g., if 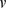 increases with temperature, cells tend to spend more time in regions of lower speed and thus aggregate at a lower temperature, i.e. 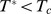 (Fig. 4B).

The shift of 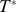 from 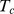 only weakly depends on 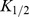, which indirectly affect 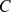 in Eq. (14) through 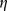 (see Eq. (8)). In fact, even the shape of the distribution 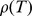 is not sensitive to 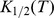, as shown in Fig. 4B. The insensitivity of thermotaxis to 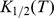 is due to the fact that 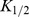 only depends on the local temperature and is the same for different cells at a given position 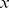, regardless of their direction of motion. In other words, 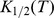 does not contribute to the tumbling rate difference 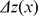 that drives the directed migration (taxis) of cells.

### Thermotaxis in Shallow Temperature Gradients: Model vs. Data

Our theory can help explain some recent experiments measuring cellular behaviors in shallow temperature gradients [36], [37]. It was observed that even the mutant bacteria lacking all chemoreceptors are still able to migrate toward high temperature [36], showing that there is an additional channel (other than sensing) in regulating bacterial thermotaxis. When the sensing channel (i.e. the bacterial signaling machinery which translates temperature stimuli into tumbling bias) does not work (e.g., due to deletion of the receptors), the temperature-dependent swimming speed can still cause the directed cell migration [36]. This is consistent with earlier work [20], [21] and our model where mutant strains lacking all receptors can be described by 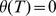 which leads to 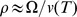 as described by Eq. (13).

**Figure 5:**
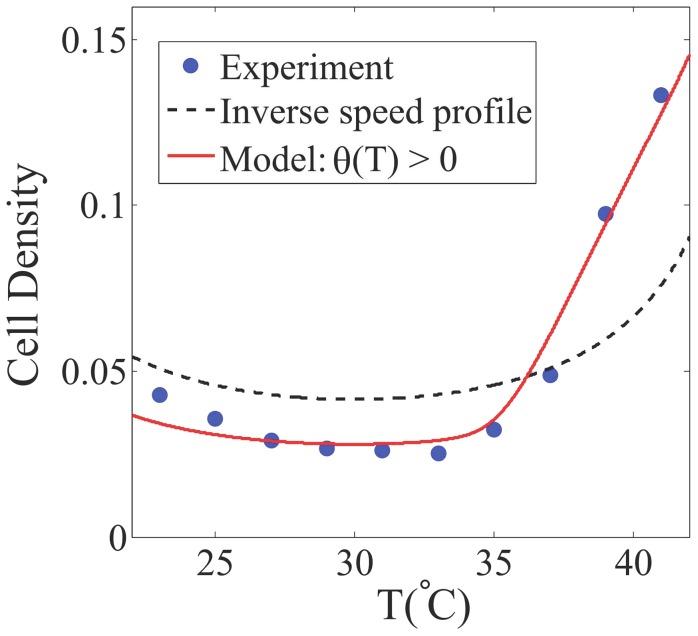
Comparison between model results and experimental data for *E. coli* thermotaxis. The blue symbols represent the cell density data obtained in Ref. [37] at 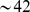 min after applying a shallow temperature gradient 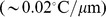. The black dashed line is the inverse speed profile, 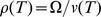 where 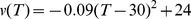 is a quadratic fitting to the measured swimming speed in Ref. [37]. The red solid line corresponds to model Eq. (13) with 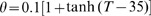 and 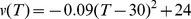. Other parameters used are the same to those in Fig. 4B.

In Ref. [37], the swimming speed 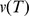 for wild-type *E. coli* (with functional chemoreceptors) was measured at different temperatures. The speed profile appears to be a quadratic function of temperature and reaches its maximum at 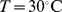. We have quantitatively compared the inverse speed profile, 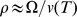, with the cell density data in Ref. [37] and found that the inverse speed profile alone could not account for the observation, especially the significant aggregation of cells at high temperatures (Fig. 5). This suggests that the thermosensory system may not be completely silent in shallow temperature gradients as suggested in [37]. We test this hypothesis by using Eq. (13) with the measured 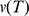 and the assumption 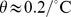 for 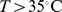. It turns out that our model, which includes both channels (speed and sensing), provides a better agreement with the observed data (Fig. 5). This result suggests that the thermosensory system may be active even in shallow temperature gradients. Further experiments are needed to examine and quantify the interplay between these two channels (speed and sensing) in shallow temperature gradients.

### Navigation under Opposing Chemical and Thermal Gradients

Our unified model can be applied to study and predict bacterial taxis behaviors in more complex environments. As a final example, we investigate the behavioral response of the Tar-only mutant cells over the interval 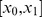 under a temperature gradient 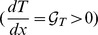 and an opposing chemoattractant gradient 
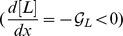
. The total free energy is modified by adding an additional ligand-depend free energy 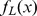 to Eq. (11). In this case, the steady-state cell density is found to be (Text S1): 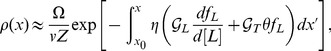
(15)where the term 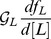 describes the chemotactic drift, and the other term 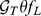 captures the interaction between the chemical and thermal signals.

**Figure 6:**
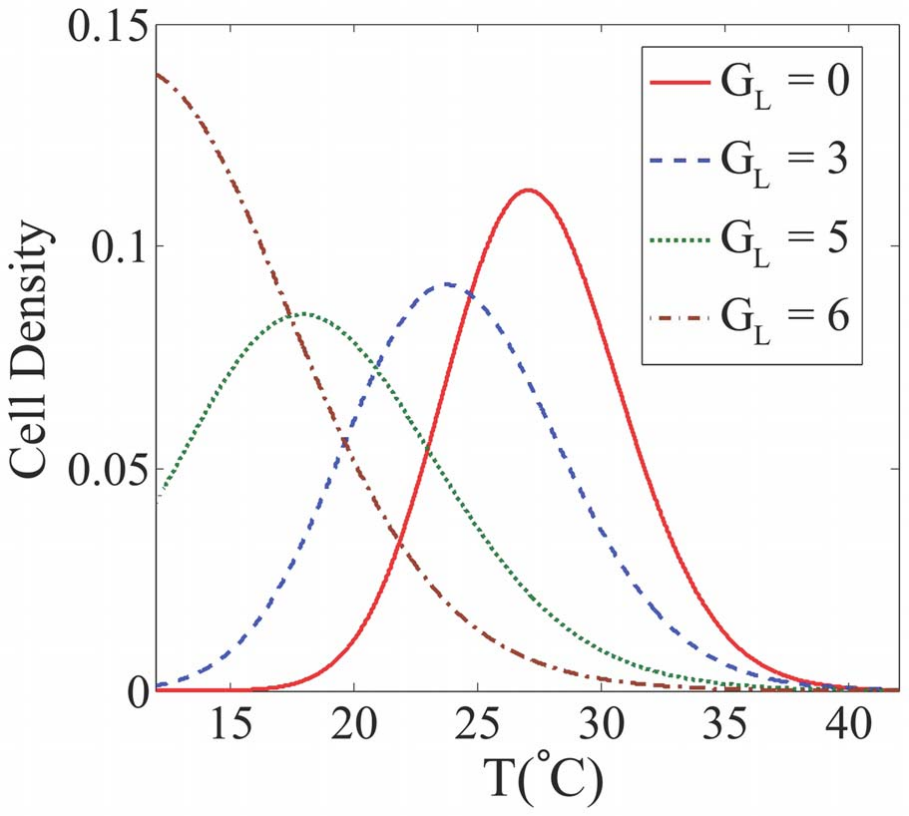
The cell density profiles under two opposing chemical and thermal gradients. The temperature gradient used here is from 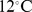 to 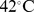 in a channel of length 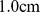. Different density profiles correspond to different attractant (MesAsp) gradients 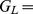
0.0, 3.0, 5.0, and 6.0 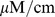 but the same concentration at the middle point: 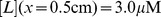. Other parameters used are the same to those in Fig. 4B.

In the absence of attractant (i.e., 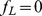), Eq. (15) recovers Eq. (13) for thermotaxis. Interestingly, for a uniform chemical background (i.e., 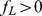 and 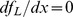), the interference effect 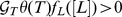 tends to suppress the accumulation of cells at high temperatures. Quantitatively, a uniform chemoattractant background can shift the preferred temperature from 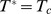 to a lower temperature 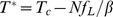. When there is an attractant gradient (so that 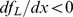) imposed against the temperature gradient, the accumulation point can be shifted further. Specifically, as the chemical gradient steepens (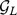 increases), the chemotacic response of bacteria become stronger, leading to a positional shift of their aggregation toward lower temperatures, as shown in Fig. 6. This example demonstrates the general capability of our model in making quantitative predictions on bacterial behaviors in complex environments (with multiple and competing chemical and nonchemical stimuli), which can be used to guide future experiments.

## Discussion

In this paper, we have incorporated the intracellular signaling pathways into the bacterial population dynamics and developed a unified model to study bacterial navigation in chemical and nonchemical gradients. This model leads to a general result, which shows that the steady state cell density is determined by the accumulative effect of the direction-dependent tumbling rates as well as the local swimming speed. In the following, we discuss some of the specific findings and related possible future directions.

### The Push-Pull Mechanism for Precision Sensing

**Figure 7:**
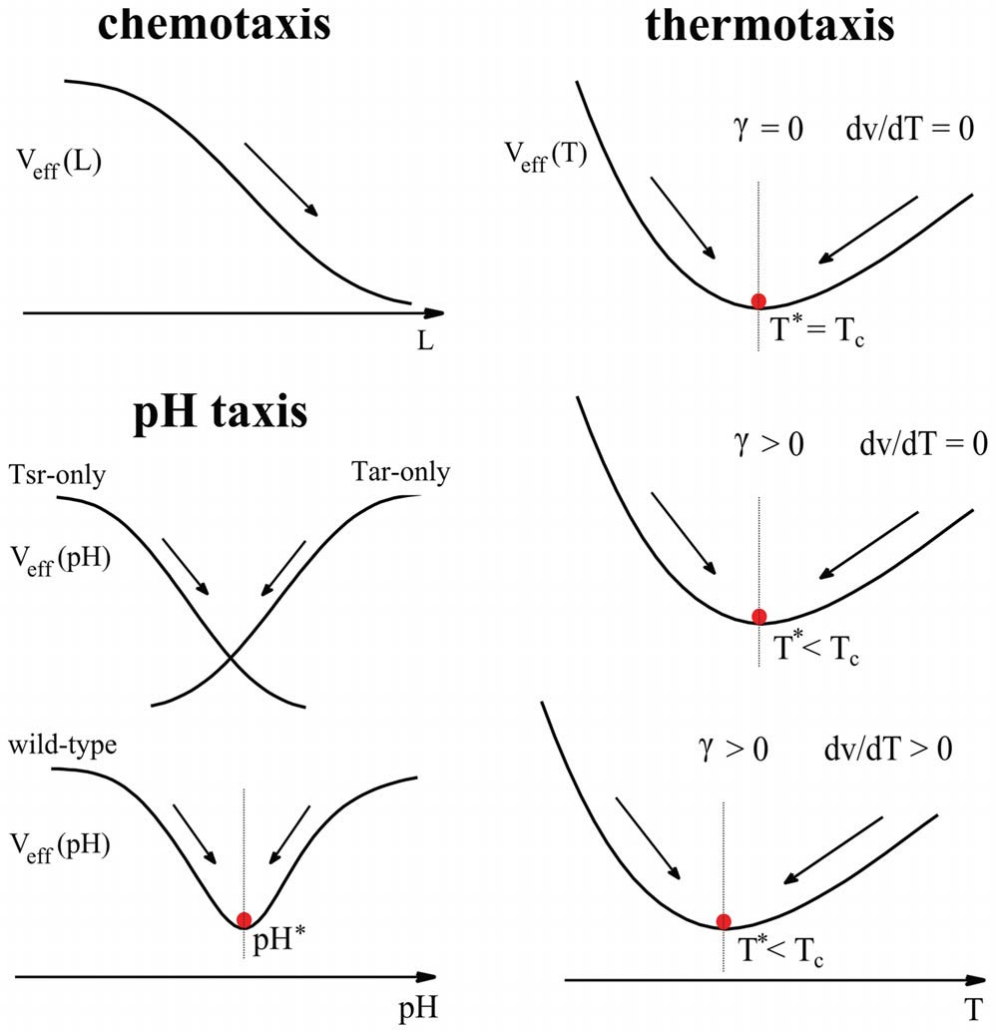
Schematic illustration of the effective potential 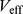 for chemotaxis, pH taxis, and thermotaxis. In the case of chemotaxis, 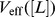 decreases monotonically as the chemoattractant concentration 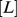 increases. For pH taxis, 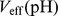 decreases with pH for Tsr-only mutant cells and increase with pH for Tar-only mutant. Based on the push-pull mechanism, 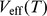 for the wild-type *E. coli* represents the balancing effect between Tar and Tsr, leading to a local minimum in the effective potential. In the case of thermotaxis, 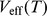 can be shifted by the effect of temperature-dependent swimming speed 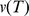. It is, however, insensitive to other temperature effects such as the temperature dependence of motor response, 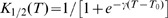, parameterized by 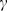.

From the population model, we can construct an effective potential function 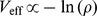, which provides a useful scheme to visualize different cases of bacterial taxis, as summarized in Fig. 7. The effective potential for chemotaxis decreases monotonically with the chemoattractant concentration and thus steer cells up the chemical gradient (Fig. 7). Our application to pH taxis illustrates how the competition between two pH sensors (Tar and Tsr) determines the preferred pH for the wild-type cells expressing both Tar and Tsr: a push-pull mechanism here creates a potential well for bacteria to accumulate (Fig. 7). In the case of *E. coli* thermotaxis, the push-pull mechanism is more subtle as the “push” and the “pull” are provided by the two sub-populations of Tar receptors with their methylation levels above or below the critical level (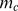). This leads to a well-defined critical temperature where cells tend to accumulate (Fig. 7). The push-pull mechanism is likely a general strategy for precision sensing. For example, it was found that two receptors, Tar and Aer, leads to a preferred level of oxygen for *E. coli* aerotaxis [38], which may also be studied within our unified model.

### Robustness and Sensitivity

*E. coli* chemotaxis has served as a model system in studying robustness of biochemical networks [34], [39], [40]. Bacteria exhibit thermal robustness in their chemotaxis network output by counterbalancing temperature effects on different opposing network components [34]. For example, the dissociation constant 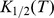 for the motor switch is observed to increase with temperature [35]. This effect balances the increase of the adapted CheY-P level with temperature such that the motor switch is able to operate in a narrow optimal range with ultrasensitivity [31]. This, however, raises a question for bacterial thermotaxis: do those temperature-sensitive factors, such as 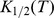 and 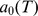, hinder the thermotactic performance? According to our model analysis, the steady state distribution of cells in a temperature gradient is mainly determined by two effects: the temperature-dependent swimming speed and the direction-dependent tumbling rates. The system is actually robust/insensitive to those instantaneous/local temperature-sensitive factors (e.g. 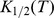) which do not contribute to the tumbling rate difference at any spatial point. The insensitivity of thermotaxis to 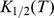, as shown from our model, is a highly desirable feature of the system as it allows robust thermotaxis without sacrificing motor-level sensitivity.

### Navigation in Complex Environments

In the natural environment, cells are often exposed to multiple chemical stimuli [41]. Our general model can be applied to study such cases (with a specific example discussed in Text S1). The density of cells subject to a multitude of chemical gradients shall be given by 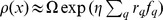, where 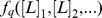 denotes the free energy contribution from all the chemical signals that are sensed by the type-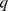 receptors. Quantitative predictions can be made about how bacterial cells integrate and respond to mixed or competitive chemical signals and how their response changes with the composition and relative abundance of their sensors. More complex situations exist when different stimuli are interdependent and/or interfere with non-chemical factors. For example, the chemical environment can be modified through consumption and secretion by the bacteria, a dynamical process depending on the bacterial cell density [36]. In addition, temperature can change the metabolic rates of bacterial cells and create temperature-dependent chemical (nutrients, oxygen) gradients. How cells navigate under such complex circumstances and how such behaviors lead to survival/growth benefits remain unclear. Our model can be extended to study those phenomena and help address those fundamental questions.

In sum, the work presented here provides a general model framework to study population behaviors in the presence of both chemical and non-chemical signals based on realistic intracellular signaling dynamics.

## Materials and Methods

Numerical simulations and figures are generated using MATLAB 7.0.

## Supporting Information

Figure S1 **Illustration of the key step in deriving the population level model.**
(PDF)

Text S1 **Technical details on the development of our unified model for bacterial taxis.**
(PDF)
