# Supplementary material for: Behaviors and Strategies of Bacterial Navigation in Chemical and Nonchemical Gradients
Source: PLoS Comput Biol. 2014 Jun 19;10(6):e1003672. doi: 10.1371/journal.pcbi.1003672 (PMC4063634; doi:10.1371/journal.pcbi.1003672)
Supplement: Figure S1 — Illustration of the key step in deriving the population level model. (PDF) [file pcbi.1003672.s001.pdf]

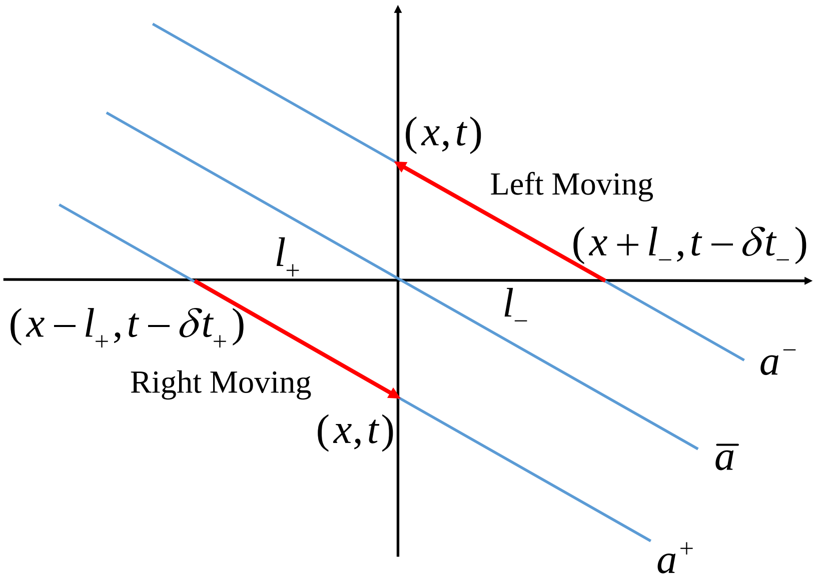

Figure S1: We imagine two scenarios: if the representative cell is moving to the right at (x, t), it may have swum along the path $(x - l_+, t - \delta t_+) \to (x, t)$ with the initial activity $a(x - l_+, t - \delta t_+) = \overline{a}(x, t)$; if the cell is moving to the left at (x, t), it may have come from the path $(x + l_-, t - \delta t_-) \to (x, t)$ with $a(x + l_-, t - \delta t_-) = \overline{a}(x, t)$.
