# Supplementary material for: Behaviors and Strategies of Bacterial Navigation in Chemical and Nonchemical Gradients
Source: PLoS Comput Biol. 2014 Jun 19;10(6):e1003672. doi: 10.1371/journal.pcbi.1003672 (PMC4063634; doi:10.1371/journal.pcbi.1003672)
Supplement: Text S1 — Technical details on the development of our unified model for bacterial taxis. (PDF) [file pcbi.1003672.s002.pdf]

Supporting Information

Behaviors and strategies of bacterial navigation in chemical and nonchemical gradients.

Bo Hu and Yuhai Tu

IBM T. J. Watson Research Center
P.O. Box 218, Yorktown Heights, NY 10598

Contents

- The Unified Model of Bacterial Taxis
- Application to Bacterial Chemotaxis under Dual Chemical Gradients
- Application to Bacterial pH Taxis
- Application to Bacterial Thermotaxis
- Bacterial Taxis under Both Chemical and Non-chemical Stimuli

The Unified Model for Bacterial Taxis

In this supplementary section, we present the analytical details about our unified model for bacterial taxis. As mentioned in the maintext, the internal state of an individual cell, represented by the receptor-kinase activity (a), can be described by the MWC model:

$$a(m,\mathbf{S}) = \frac{1}{1+\exp[Nf_a(m,\mathbf{S})]}. \tag{S1}$$

where $\mathbf{S}(x) = \{S_1, S_2, ...\}$ represents the external condition and m denotes the average (aggregate) methylation level of the receptor cluster. The receptor methylation-demethylation process takes place in a slow time-scale, τ_a, and restores the total activity, a, to the adapted level, a_0. Such receptor-modification reactions serves as the cell's memory about the environment and can be described by the following differential equation:

$$\frac{dm}{dt} = \frac{a_0(\mathbf{S}) - a(m,\mathbf{S})}{\tau_a(\mathbf{S})} \tag{S2}$$

The receptor activity, a, regulates the level of the intracellular response regulator (CheY-P) which promotes the clockwise (CW) rotation of motors. A swimming cell tumbles when its motors rotate clockwise or when it experiences rotational diffusion (denoted by z_0). The total tumbling rate is

$$z(a) = z_0 + z_1(a) = z_0 + \tau^{-1}(a/K_{1/2})^H. \tag{S3}$$

Here, $1/\tau$ sets the duration time of the tumbling state, $K_{1/2}$ represents the activity level at which the CW bias is 0.5, and H denote the Hill coefficient of the motor response function, respectively. In the one-dimensional setup, a cell moves toward either the right (+) or the left ($-$) direction with speed $v(\mathbf{S})$ and changes its direction randomly with the tumbling frequency $z(a)$.

In response to the environmental stimuli, an ensemble of bacterial cells will migrate in the physical space and also distribute in the internal state space (due to memory). We define $P^{\pm}(x,a,t)$ as the probability density of cells which is in state a and move in the "$\pm$" direction at (x,t). The master equation governing $P^{\pm}(x,a,t)$ is

$$\frac{\partial P^+}{\partial t} = -\frac{\partial[G^+(a,\mathbf{S})P^+]}{\partial a} - \frac{\partial[v(\mathbf{S})P^+]}{\partial x} - \frac{z(a)}{2}(P^+ - P^-), \tag{S4}$$

$$\frac{\partial P^-}{\partial t} = -\frac{\partial[G^-(a,\mathbf{S})P^-]}{\partial a} + \frac{\partial[v(\mathbf{S})P^-]}{\partial x} + \frac{z(a)}{2}(P^+ - P^-), \tag{S5}$$

where $G^{\pm}(a,\mathbf{S})$ are the rate functions for the internal state variable a as the cell moves in the "$\pm$" direction. Since a is a function of m and $\mathbf{S}$, its

change over time is contributed by the local methylation changes and the cell migration in space, i.e.,

$$\frac{da}{dt} = G^{\pm}(a, \mathbf{S}) = \frac{\partial a}{\partial m}\frac{dm}{dt} \pm \frac{\partial a}{\partial \mathbf{S}}\frac{d\mathbf{S}}{dt} = \frac{\partial a}{\partial m}\frac{a_0 - a}{\tau_a} \pm \frac{\partial a}{\partial \mathbf{S}} \cdot \frac{d\mathbf{S}}{dx} v. \tag{S6}$$

We define the density (ρ) and the flux (J) of cells in the spatial coordinate:

$$\rho(x,t) = \int \left[P^+(x,a,t) + P^-(x,a,t)\right] da \tag{S7}$$

$$J(x,t) = \int v \left[P^+(x,a,t) - P^-(x,a,t)\right] da. \tag{S8}$$

Summing Eqs. (S4) and (S5) and integrating over a leads to

$$\frac{\partial \rho}{\partial t} = -\frac{\partial J}{\partial x}. \tag{S9}$$

Similarly, subtracting these two equations and integrating over a yields

$$\frac{1}{v(\mathbf{S})}\frac{\partial J}{\partial t} = -\frac{\partial [v(\mathbf{S})\rho]}{\partial x} - \int z(a)(P^+ - P^-)da. \tag{S10}$$

The system over a finite region $[x_0, x_1]$ with reflecting boundaries should satisfy the zero flux condition (i.e., $J = 0$) in steady state. Thus we should have $\int P^+ da = \int P^- da = \rho(x)/2$ in steady state and Eq. (S10) becomes

$$-\frac{\partial [v(\mathbf{S})\rho]}{\partial x} = \int z(a)(P^+ - P^-)da = \frac{[z^+(x) - z^-(x)]\rho}{2} = \Delta z \rho. \tag{S11}$$

where $z^{\pm}(x) = \int z P^{\pm} da / \int P^{\pm} da$ represents the average tumbling rate for the right or left moving cells at position x. From Eq. (S11), one can easily find the equilibrium cell density:

$$\rho(x, t \to \infty) = \frac{\Omega}{v(x)} \exp\left[-\int_{x_0}^{x} \frac{\Delta z(x')}{v(x')} dx'\right]. \tag{S12}$$

For the simple case where both v and Δz are (nonzero) constant, the steady-state cell density takes an exponential profile in space. Here, the chemotactic drift arises purely from the tumbling rate difference between the left and right moving populations. Intuitively, if $z^+(x) < z^-(x)$ for example, then on average cells tend to move in the right (+) direction because it is more difficult for cells to enter a region where they tend to tumble more frequently.

The origin of Δz is that the average activity over the left-moving cells ($a^+ = \frac{\int aP^+ da}{\int P^+ da}$) differs from the average over the right-moving population ($a^- = \frac{\int aP^- da}{\int P^- da}$). The average receptor activity in the steady state is

$$\overline{a}(x) \equiv \frac{1}{\rho}\int a(P^+ + P^-)da = \frac{a^+ + a^-}{2}. \tag{S13}$$

We can estimate the activity difference $(a^+ - a^-) \equiv \Delta a$ by considering the scenario in Fig. S1: If the representative cell is moving to the right at (x,t), it may have swum all the way along the path $(x - l_+, t - \delta t_+) \to (x,t)$ with the initial activity $a^+(x - l_+, t - \delta t_+) = \overline{a}(x,t)$; if the cell is moving to the left at (x,t), it may have come from the path $(x + l_-, t - \delta t_-) \to (x,t)$ with $a^-(x + l_-, t - \delta t_-) = \overline{a}(x,t)$. Let $G^{\pm}(a, \mathbf{S}) = G_m(a, \mathbf{S}) + G_S^{\pm}(a, \mathbf{S})$ where

$$G_m(a, \mathbf{S}) = \frac{\partial a}{\partial m} \cdot \frac{a_0(\mathbf{S}) - a(m, \mathbf{S})}{\tau_a(\mathbf{S})}, \quad \text{and} \quad G_S^{\pm}(a, \mathbf{S}) = \pm\frac{\partial a}{\partial \mathbf{S}} \cdot \frac{d\mathbf{S}}{dx} v(\mathbf{S}). \tag{S14}$$

Then, we have the following approximations along the two paths:

$$\begin{aligned} a^+(x,t) - a^+(x - l_+, t - \delta t_+) &= a^+(x,t) - \overline{a}(x,t) \approx G^+(\overline{a}(x,t), \mathbf{S}(x - l_+))\delta t_+, \\ a^-(x,t) - a^+(x + l_-, t - \delta t_-) &= a^-(x,t) - \overline{a}(x,t) \approx G^-(\overline{a}(x,t), \mathbf{S}(x + l_-))\delta t_-. \end{aligned}$$

Let $\mathbf{S}(x) - \mathbf{S}(x - l_+) \equiv \delta\mathbf{S}_+ \approx \frac{d\mathbf{S}}{dx} v \delta t_+$ and $\mathbf{S}(x + l_-) - \mathbf{S}(x) \equiv \delta\mathbf{S}_- \approx \frac{d\mathbf{S}}{dx} v \delta t_-$. Since the direction of motion is randomized during each tumbling event, the time scale of δt_+ and δt_- (durations of swimming without tumbling) is set by the short run time $1/z(\overline{a})$. Then one can make Taylor expansions around $\mathbf{S}(x)$ and derive the difference between a^+ and a^- at (x,t):

$$\begin{aligned} 2\Delta a(x,t) &\equiv a^+ - a^- \approx \frac{G^+(\overline{a}, \mathbf{S}(x) - \delta\mathbf{S}_+) - G^-(\overline{a}, \mathbf{S}(x) + \delta\mathbf{S}_-)}{z(\overline{a})} \\ &= \frac{G_m(\overline{a}, \mathbf{S}(x) - \delta\mathbf{S}_+) - G_m(\overline{a}, \mathbf{S}(x) + \delta\mathbf{S}_-)}{z(\overline{a})} \\ &\quad + \frac{G_S^+(\overline{a}, \mathbf{S}(x) - \delta\mathbf{S}_+) - G_S^-(\overline{a}, \mathbf{S}(x) + \delta\mathbf{S}_-)}{z(\overline{a})} \\ &\approx -\left[\frac{\partial G_m}{\partial \mathbf{S}}(\overline{a}, \mathbf{S})\right] \frac{\delta\mathbf{S}_+ + \delta\mathbf{S}_-}{z(\overline{a})} + \frac{G_S^+(\overline{a}, \mathbf{S}) - G_S^-(\overline{a}, \mathbf{S})}{z(\overline{a})} \\ &\quad - \left[\frac{\partial G_S^+}{\partial \mathbf{S}}(\overline{a}, \mathbf{S})\right] \frac{\delta\mathbf{S}_+}{z(\overline{a})} - \left[\frac{\partial G_S^-}{\partial \mathbf{S}}(\overline{a}, \mathbf{S})\right] \frac{\delta\mathbf{S}_-}{z(\overline{a})} \\ &\approx -\frac{2v(\mathbf{S})}{z(\overline{a})} \left[\frac{1}{z(\overline{a})} \frac{\partial G_m}{\partial \mathbf{S}}(\overline{a}, \mathbf{S})\right] \frac{d\mathbf{S}}{dx} \\ &\quad + \frac{2v(\mathbf{S})}{z(\overline{a})} \left[\frac{\partial a}{\partial \mathbf{S}}(\overline{a}, \mathbf{S})\right] \frac{d\mathbf{S}}{dx} + \mathcal{O}\left(\frac{d\mathbf{S}}{dx}\right)^2. \end{aligned} \tag{S15}$$

It is worth remarking that the function G_m has a slow time scale set by the intrinsic methylation time scale τ_a which is much longer than the average run time $z^{-1} \sim 1s$. This suggests that in the last line of Eq. (S15) we have

$$\frac{1}{z}\frac{\partial G_m}{\partial \mathbf{S}}(\overline{a}, \mathbf{S}) \propto \frac{z^{-1}}{\tau_a} \tag{S16}$$

which becomes negligible given $\tau_a \gg z^{-1}$. Therefore, we arrive at the following key approximation for Δa:

$$\Delta a \approx \frac{v(\mathbf{S})}{z(\overline{a})}\frac{d\mathbf{S}}{dx}\left(\frac{\partial a}{\partial \mathbf{S}}\right)_{a=\overline{a}} \tag{S17}$$

Then we can use the approximation $\Delta z \approx \left(\frac{\partial z}{\partial a}\right)_{a=\overline{a}}\Delta a$ and Eq. (S17) to rewrite Eq. (S12) as follows:

$$\rho(x) \approx \frac{\Omega}{v(\mathbf{S})}\exp\left[-\int_{x_0}^{x}\left(\frac{\partial \ln z}{\partial a}\cdot\frac{\partial a}{\partial \mathbf{S}}\right)_{a=\overline{a}} d\mathbf{S}\right]. \tag{S18}$$

Eq. (S18) shows that the equilibrium distribution is ultimately shaped by the two motility behaviors, tumbling (z) and swimming (v), both of which directly or indirectly depend on the external conditions. To complete this model, we still need the dynamic equation for $\overline{a}$. First, we notice the following

$$\frac{\partial(\overline{a}\rho)}{\partial t} = \frac{\partial}{\partial t}\left(a^+\int P^+ da + a^-\int P^- da\right) = \int \frac{\partial}{\partial t}(aP^+ + aP^-)da. \tag{S19}$$

Then by using Eqs. (S4) and (S5), we find that

$$\begin{aligned}\frac{\partial(\overline{a}\rho)}{\partial t} &= -\frac{\partial}{\partial x}\int va(P^+ - P^-)da - \int a\frac{\partial}{\partial a}(G^+P^+ + G^-P^-)da \\ &\approx -\frac{\partial}{\partial x}(\overline{a}J + \rho v\Delta a) + \int (G^+P^+ + G^-P^-)da. \end{aligned}\tag{S20}$$

Using Eq. (S17), one can see that $\overline{a}(x)$ in steady state ($J = 0$) should satisfy:

$$\frac{\partial}{\partial x}\left[\frac{\rho v^2}{z(\overline{a})}\frac{d\mathbf{S}}{dx}\left(\frac{\partial a}{\partial \mathbf{S}}\right)_{a=\overline{a}}\right] = \int (G^+P^+ + G^-P^-)da, \tag{S21}$$

where, to the first order approximation in the shallow gradients, we have

$$\begin{aligned}\int (G^+P^+ + G^-P^-)da &= \int \frac{\partial a}{\partial m}\frac{a_0 - a}{\tau_a}(P^+ + P^-)da + \int \frac{\partial a}{\partial \mathbf{S}}\frac{d\mathbf{S}}{dx}v(P^+ - P^-)da \\ &\approx \left(\frac{\partial a}{\partial m}\frac{a_0 - a}{\tau_a}\rho\right)_{a=\overline{a}}.\end{aligned}$$

Thus, Eq. (S21) is equivalent to

$$\frac{\partial}{\partial x}\left[\frac{v^2}{z(\overline{a})}\frac{d\mathbf{S}}{dx}\left(\frac{\partial a}{\partial \mathbf{S}}\right)_{a=\overline{a}}\right]+\frac{\partial \ln\rho}{\partial x}\left[\frac{v^2}{z(\overline{a})}\frac{d\mathbf{S}}{dx}\left(\frac{\partial a}{\partial \mathbf{S}}\right)_{a=\overline{a}}\right]\approx\left(\frac{\partial a}{\partial m}\frac{a_0-a}{\tau_a}\right)_{a=\overline{a}}. \quad \text{(S22)}$$

Since $\frac{\partial \ln\rho}{\partial x}\propto\frac{d\mathbf{S}}{dx}$ and $\frac{\partial}{\partial x}=\frac{d\mathbf{S}}{dx}\frac{\partial}{\partial \mathbf{S}}$, Eq. (S22) implies that

$$\left(\frac{\partial a}{\partial m}\frac{a_0-a}{\tau_a}\right)_{a=\overline{a}}\approx 0+\mathcal{O}\left(\frac{d\mathbf{S}}{dx}\right)^2. \quad \text{(S23)}$$

Given $\frac{\partial a}{\partial m}\neq 0$, Eq. (S23) indicates that $\overline{a}(x)\approx a_0$ in shallow gradients. If one keeps the second-order term in Eq. (S22), one will see that the average activity $\overline{a}(x)$ tends to increase with the gradient steepness.

Application to Bacterial Chemotaxis under Dual Chemical Gradients

In the natural environment, cells are often exposed to multiple chemical stimuli. Here we extend our analysis to the case where bacterial cells respond to two opposing chemoattractant gradients (aspartate: $\frac{d[L]_1}{dx}>0$ and serine: $\frac{d[L]_2}{dx}<0$) that are sensed by the two most abundant *E. coli* receptors, Tar and Tsr, respectively. The free energy energy for the receptor-kinase activity in the MWC model is given by:

$$\begin{aligned}f_a(m,[L]_1,[L]_2) &= f_m(m)+r_1 f_{L1}([L]_1)+r_2 f_{L2}([L]_2)\\ &= -E_m(m-m_0)+r_1\ln\frac{1+[L]_1/K_1^I}{1+[L]_1/K_1^A}+r_2\ln\frac{1+[L]_2/K_2^I}{1+[L]_2/K_2^A}. \quad \text{(S24)}\end{aligned}$$

The steady-state cell density is found to be

$$\rho(x)\approx\Omega e^{\eta(r_1 f_{L1}+r_2 f_{L2})}, \quad \text{(S25)}$$

Clearly, the density profile ρ depends on the Tar/Tsr ratio (r_1/r_2). When Tar (or Tsr) dominates in the receptor complex, cells are expected to respond mainly to aspartate (or serine) gradient. For intermediate values of r_1/r_2, the competition between Tar and Tsr may lead to the accumulation of cells around some position x^*. This position x^* can be determined by the first-order condition $V'_{\text{eff}}(x^*)=0$, where $V_{\text{eff}}=-\eta(r_1 f_{L1}+r_2 f_{L2})$. Define $\mathcal{G}_{1,2}=|d[L]_{1,2}/dx|$. Then, in the logarithmic sensing regime, the first order condition suggests that $r_1\mathcal{G}_1/[L]_1(x^*)\approx r_2\mathcal{G}_2/[L]_2(x^*)$. If the solution of x^* exists within the interval $[x_0,x_1]$, then it is given by

$$x^*\approx\frac{\mathcal{G}_1 r_1[L]_2(x_0)-\mathcal{G}_2 r_2[L]_1(x_0)}{\mathcal{G}_1\mathcal{G}_2(r_1+r_2)}=r_1\frac{[L]_2(x_0)}{\mathcal{G}_2}-r_2\frac{[L]_1(x_0)}{\mathcal{G}_1}. \quad \text{(S26)}$$

Application to Bacterial pH Taxis

In this supplementary section, we consider the case of bacterial pH taxis. The free energy for the total receptor-kinase activity is

$$f_a(m, \mathrm{pH}) = f_m + r_1 f_1(\mathrm{pH}) + r_2 f_2(\mathrm{pH}) = f_m + \sum_{q=1,2} r_q \ln \frac{1 + 10^{K_q^I - \mathrm{pH}}}{1 + 10^{K_q^A - \mathrm{pH}}}. \quad \text{(S27)}$$

The steady-state cell distribution in a pH gradient: $\rho(x) \approx \Omega \exp[-V_{\text{eff}}(x)]$, with the effective potential $V_{\text{eff}} = -\eta(r_1 f_1 + r_2 f_2)$. We can use the first-order condition, $V'_{\text{eff}}(\mathrm{pH}^*) = -\eta[r_1 f'_1(\mathrm{pH}^*) + r_2 f'_2(\mathrm{pH}^*)] = 0$, to determine the preferred pH. Let $w^* \equiv 10^{\mathrm{pH}^*}$, $w_q^A \equiv 10^{K_q^A}$ and $w_q^I \equiv 10^{K_q^I}$ for $q = 1, 2$, we find that

$$\frac{r_1}{r_2} = -\frac{f'_2(\mathrm{pH}^*)}{f'_1(\mathrm{pH}^*)} = \frac{w_2^A - w_2^I}{w_1^I - w_1^A} \cdot \frac{(w^* + w_1^A)(w^* + w_1^I)}{(w^* + w_2^A)(w^* + w_2^I)}, \quad \text{(S28)}$$

which is simply a quadratic equation of w^*. The observed opposite responses to pH changes indicate that $K_1^A < K_1^I$ for Tar and $K_2^A > K_2^I$ for Tsr. Thus, we expect that $w_1^I \gg w_1^A$, $w_2^A \gg w_2^I$, and $w_1^I \gg w^* \gg w_2^I$, which together simplify Eq. (S28) as

$$\frac{r_1}{r_2} \approx \frac{w_2^A}{w_1^I} \cdot \frac{(w^* + w_1^A)}{(w^* + w_2^A)} \cdot \frac{w_1^I}{w^*} = \frac{w_2^A}{w^*} \cdot \frac{(w^* + w_1^A)}{(w^* + w_2^A)}, \quad \text{(S29)}$$

Therefore, for a given Tar/Tsr ratio (r_1/r_2), the preferred pH is mostly determined by the values of w_1^A and w_2^A (i.e., K_1^A and K_2^A). The exact solution to Eq. (S29) is given by

$$w^* = \frac{w_2^A \left(1 - \frac{r_1}{r_2}\right) + w_2^A \sqrt{\left(1 - \frac{r_1}{r_2}\right)^2 + 4\frac{r_1 w_1^A}{r_2 w_2^A}}}{2(r_1/r_2)}. \quad \text{(S30)}$$

For the special case that $r_1 = r_2$, Eq. (S29) has a simple solution, $w^* = \sqrt{w_1^A w_2^A}$, which means $\mathrm{pH}^* = (K_1^A + K_2^A)/2$ at $r_1/r_2 = 1$. Considering the simple scenario where $K_1^I > K_1^A = K_2^A > K_2^I$ (i.e., $w_1^I \gg w_1^A = w_2^A \gg w_2^I$), we can obtain from Eq. (S29) that $w^* \approx w_2^A r_2/r_1$ and correspondingly the preferred pH level is

$$\mathrm{pH}^* = K_2^A - \log_{10}(r_1/r_2). \quad \text{(S31)}$$

Thus, we can conjecture an empirical relationship between pH^* and r_1/r_2:

$$\mathrm{pH}^* \approx \frac{K_1^A + K_2^A}{2} - \lambda \log_{10}\left(\frac{r_1}{r_2}\right), \quad \text{(S32)}$$

where λ represents the sensitivity of pH* to the change of the Tar/Tsr ratio. The parameter λ depends on the relative values of K_1^A and K_2^A, and can be calculated numerically. The empirical Eq. (S32) has been tested through extensive numerical experiments and turns to be useful for our data analysis.

Application to Bacterial Thermotaxis

In this supplementary section, we apply our unified model to bacterial thermotaxis. The free energy for the Tar receptor activity is assumed to be

$$f_a(m,T) \approx -E_m(m-m_0) - (m-m_c)\cdot g(T). \tag{S33}$$

Here, $g(T)$ satisfies $g'(T) > 0$ and $g(T_0) = 0$ at a reference temperature T_0 where E_m is determined ($-\frac{\partial f_a}{\partial m}|_{T=T0} = E_m$). By Eq. (S33), one can find that

$$\frac{\partial a}{\partial T} = -Na(1-a)\frac{\partial f_a}{\partial T} = Na(1-a)(m-m_c)\frac{dg(T)}{dT}. \tag{S34}$$

Thus, consistent with the experiment, the Tar receptor in our model acts as a warm sensor ($\frac{\partial a}{\partial T} < 0$) when $m < m_c$ and as a cold sensor ($\frac{\partial a}{\partial T} > 0$) sensor when $m > m_c$. The (steady-state) adapted activity depends on temperature and can be modeled as:

$$a_0(T) = \frac{k_R(T)}{k_R(T)+k_B(T)} \approx \frac{1}{1+\exp[-\beta(T-T_0)]}. \tag{S35}$$

It is observed that $a_0 \approx 1/3$ at room temperature $T = 22°$C and $a_0 \approx 1/2$ at $T_0 = 32°$C, from which we estimate that $\beta \approx 0.07/°$C. The critical temperature T_c at which the Tar receptors invert response is set by $\frac{\partial a}{\partial T}|_{T=T_c} = 0$ which is equivalent to $m(T_c) = m_c$ by Eq. (S34). The steady state methylation level $m(T)$ can be determined by $dm/dt = 0$ or $a(m,T) = a_0(T)$. It amounts to solve $Nf_a(m,T) = -\beta(T-T_0)$ which yields

$$m(T) = \frac{m_c g(T) + \beta(T-T_0)/N + E_m m_0}{g(T)+E_m}. \tag{S36}$$

Thus, using Eq. (S36) in the condition $m(T_c) = m_c$, one can find

$$T_c = T_0 + \frac{NE_m(m_c-m_0)}{\beta}, \tag{S37}$$

which shows how the critical temperature is encoded in the signaling pathway. Combining Eq. (S36) and Eq. (S37), one can show that

$$m(T) - m_c = \frac{\beta(T-T_0)/N + E_m(m_0-m_c)}{g(T)+E_m} = \frac{\beta(T-T_c)}{N[g(T)+E_m]}, \tag{S38}$$

which changes sign at T_c given that $g(T) + E_m > 0$ holds for the range of temperature under consideration.

By Eq. (S18), the steady-state cell distribution in a linear temperature gradient over the physiological range $[T_-, T_+]$ can be calculated as follows

$$\begin{aligned}\rho(T) &\approx \frac{\Omega}{v(T)} \exp\left[-\int_{T_-}^{T} \left(\frac{\partial \ln z}{\partial \hat{T}} \right)_{a=\bar{a}} d\hat{T} \right] \\ &= \frac{\Omega}{v(T)} \exp\left\{ -\int_{T_-}^{T} \eta(\hat{T})[m(\hat{T}) - m_c] g'(\hat{T}) d\hat{T} \right\} \\ &= \frac{\Omega}{v(T)} \exp\left\{ -\frac{\beta}{N} \int_{T_-}^{T} \frac{(\hat{T} - T_c)\eta(\hat{T}) g'(\hat{T})}{g(\hat{T}) + E_m} d\hat{T} \right\}. \end{aligned} \tag{S39}$$

In deriving Eq. (S39), we have used Eq. (S34) and Eq. (S38). The expression of $\rho(T)$ is composed of two parts: the temperature-dependent swim speed $v(T)$ and the temperature-dependent effect from the tumbling behavior. From Eq. (S39), we can see that cells are able to accumulate near the critical temperature T_c as long as $\beta > 0$, $g'(T) > 0$, and $g(T) + E_m > 0$. The first condition $\beta > 0$ reflects the temperature-dependent imperfect adaptation kinetics for *E. coli* and is required for the existence of the critical temperature; see Eq. (S37). The second condition $g'(T) > 0$ ensures that Tar acts as a warm sensor when its methylation level m is below m_c and switches to a cold sensor when $m > m_c$; see Eq. (S34). The third condition $g(T) + E_m > 0$ means that an increase of the methylation level will always reduce the free energy in Eq. (S33) and ensures that the methylation level $m(T)$ increases with the temperature near T_c; see Eq. (S38).

For convenience, we can define a general temperature-dependent function:

$$\theta(T) = \frac{g'(T)}{g(T) + E_m}, \tag{S40}$$

Since $g'(T_0) = 0$, the above equation determines the following transformation

$$g(T) = E_m \left[\exp\left(\int_{T_0}^{T} \theta(\hat{T}) d\hat{T} \right) - 1 \right], \tag{S41}$$

which allows us to rewrite the free energy Eq. (S33) as follows

$$f_a(m, T) = -E_m(m_c - m_0) - E_m(m - m_c) \exp\left[\int_{T_0}^{T} \theta(\hat{T}) d\hat{T} \right]. \tag{S42}$$

Thus, $\theta(T)$ determines $g(T)$ and contains all the information about how the receptor activity depends on temperature. We further introduce

$$Z(T) \equiv \exp\left\{ \frac{\beta}{N} \int_{T_-}^{T} (\hat{T} - T_c)\eta(\hat{T})\theta(\hat{T}) d\hat{T} \right\}, \tag{S43}$$

which represents the accumulative effect of the tumbling rate difference Δz on the cell migration. Using Eqs. (S40) and (S43), we rewrite Eq. (S39) as

$$\rho(T) \approx \frac{\Omega}{v(T)} \exp\left\{-\frac{\beta}{N}\int_{T_-}^{T} (\hat{T}-T_c)\eta(\hat{T})\theta(\hat{T})d\hat{T}\right\} = \frac{\Omega}{v(T)Z(T)}. \quad \text{(S44)}$$

Thus, the effective potential function for bacterial thermotaxis is

$$V_{\text{eff}}(T) = \ln v(T) + \frac{\beta}{N}\int_{T_-}^{T} (\hat{T}-T_c)\eta(\hat{T})\theta(\hat{T})d\hat{T}. \quad \text{(S45)}$$

From the condition $V'_{\text{eff}}(T^*) = 0$, we can find the preferred temperature

$$T^* = T_c - \frac{N}{\beta\eta(T^*)\theta(T^*)}\left(\frac{\partial \ln v}{\partial T}\right)_{T=T^*}. \quad \text{(S46)}$$

Thus, if v is constant, the first-order condition gives that $T^* = T_c$ and the second-order condition, $V''_{\text{eff}}(T^*) = \frac{\beta}{N}\eta(T^*)\theta(T^*)$, indicates that $T^* = T_c$ is the preferred temperature only if $\theta(T) > 0$. If, however, the swimming speed v increases with T, then Eq. (S46) suggests that $T^* < T_c$. Last, for mutant cells lacking all receptors ($\theta(T) = 0$), the density becomes $\rho(T) = \Omega/v(T)$ and T^* corresponds to the temperature where $v(T^*)$ reaches its minimum.

Bacterial Taxis under Both Chemical and Non-Chemical Stimuli

Our unified model can be applied to study bacterial taxis in more complex environments. As a final example, we investigate the bacterial response to an integration of both chemical and nonchemical stimuli. Specifically, we consider the decision-making process of the Tar-only mutant cells under two opposing linear gradients over the interval $[x_0, x_1]$: a temperature gradient ($\frac{dT}{dx} = \mathcal{G}_T > 0$) and a chemoattractant gradient ($\frac{d[L]}{dx} = -\mathcal{G}_L < 0$). Both signals are sensed by Tar whose receptor-kinase activity is given by $a = 1/\left[1 + e^{Nf_a(m,[L],T)}\right]$, with the free energy difference between two states,

$$f_a(m,[L],T) \approx -E_m(m-m_0) - (m-m_c)\cdot g(T) + f_L. \quad \text{(S47)}$$

Here, $f_L(x) \equiv \ln\frac{1+[L](x)/K_I}{1+[L](x)/K_A}$ represents the ligand-dependent free energy which increases with $[L]$ and hence decreases with x. The steady-state methylation level is determined by $dm/dt = 0$ or $a(m,[L],T) = a_0(T)$. This is equivalent to $Nf_a(m,[L],T) = -\beta(T-T_0)$, from which one can solve

$$m([L],T) = \frac{m_c g(T) + \beta(T-T_0)/N + E_m m_0 + f_L([L])}{g(T)+E_m} = m(x), \quad \text{(S48)}$$

In the presence of a chemical gradient, the critical temperature at which Tar inverts response is no longer defined intrinsically because it depends on the external chemical condition. However, we can still use Eq. (S37) which defines T_c as an intrinsic parameter (solely encoded by the signal transduction pathway). To avoid confusion, T_c in this section always refers to Eq. (S37) and is not necessarily the critical temperature for Tar to change its sensing mode. Then we can use Eq. (S37) to rewrite Eq. (S48) as follows

$$m(x) - m_c = \frac{\beta[T(x) - T_c]/N + f_L(x)}{g(T(x)) + E_m}. \tag{S49}$$

Since f_L decreases with x, it is possible that the steady-state methylation level m also decreases with x if the chemical gradient is strong enough. For appropriate gradient profiles of $[L](x)$ and $T(x)$, there may exist a particular position x_c satisfying $m(x_c) = m_c$. Then, by Eq. (S49) we find that

$$T(x_c) = T_c - \frac{N f_L(x_c)}{\beta}, \tag{S50}$$

from which x_c can be determined, although the solution may be out of the interval $[x_0, x_1]$ where cells are constrained.

Again, we use Eq. (S18) to derive the steady-state cell distribution:

$$\begin{aligned} \rho(x) &\approx \frac{\Omega}{v(T)} \exp\left[-\int_{x_0}^{x} \frac{dT}{dx'}\left(\frac{\partial \ln z}{\partial T}\right)_{a=a_0} dx' - \int_{x_0}^{x} \frac{d[L]}{dx'}\left(\frac{\partial \ln z}{\partial [L]}\right)_{a=a_0} dx'\right] \\ &= \frac{\Omega}{v(T)} \exp\left\{-\int_{x_0}^{x} \eta(x')\left[\mathcal{G}_T[m(x') - m_c]\frac{dg}{dT}(x') + \mathcal{G}_L\frac{df_L}{d[L]}(x')\right] dx'\right\} \\ &= \frac{\Omega}{v \cdot Z} \exp\left\{-\int_{x_0}^{x} \eta(x')\left[\mathcal{G}_T f_L(x')\theta(T(x')) + \mathcal{G}_L\frac{df_L}{d[L]}(x')\right] dx'\right\}. \end{aligned} \tag{S51}$$

Clearly, without any chemical interference (i.e., $f_L = 0$), Eq. (S51) recovers our earlier result Eq. (S44) for thermotaxis. For uniform chemical background ($f_L > 0$ and $df_L/dx = 0$), Eq. (S51) becomes

$$\rho(T) \approx \frac{\Omega}{v(T)Z(T)} \exp\left[-f_L \int_{T_-}^{T} \eta(\hat{T})\theta(\hat{T})d\hat{T}\right]. \tag{S52}$$

Compared to Eq. (S44), the extra exponential term in Eq. (S52) is a decreasing function of T if $\theta(T) > 0$. This will suppress the accumulation of cells at high temperatures. For constant v, it is easy to verify that the presence of a uniform chemoattractant background can shift the preferred temperature from $T^* = T_c$ to a lower temperature $T^* = T_c - N f_L/\beta$, in agreement with Eq. (S50). Now, if we increase the chemical gradient ($df_L/dx < 0$) against the temperature gradient, more and more cells will be lured away to chase the chemoattractant.
